# A public private partnership to fight against malaria along the Chad-Cameroon pipeline corridor: I. Baseline data on socio-anthropological aspects, knowledge, attitudes and practices of the population concerning malaria

**DOI:** 10.1186/1471-2458-13-1023

**Published:** 2013-10-29

**Authors:** Roger Moyou-Somo, Paul Essomba, Eva Songue, Natacha Nsiewe Tchoubou, Anita Ntambo, Huguette Ngo Hiol, Jacques Pokam Kemajou, Marie-José Essi, Pascal Millet

**Affiliations:** 1 Department of Microbiology, Hematology, Parasitology & Infectious Diseases, Faculty of Medicine & Biomedical Sciences, University of Yaoundé I, Yaoundé, Cameroon; 2 Institute of Medical Research and Medicinal Plants Studies (IMPM), Yaoundé, Cameroon; 3 University of Bordeaux 2, Victor Ségalen, 146 rue Leo Saignat, 33076, Bordeaux, France; 4 East Regional Hospital, Bertoua, Cameroon; 5 Department of Public Health, Faculty of Medicine & Biomedical Sciences, University of Yaoundé I, Yaoundé, Cameroon; 6 National Institute of Cartography, Yaoundé, Cameroon

**Keywords:** Malaria, Chad-Cameroon pipeline, Knowledge, Attitudes and practices concerning malaria

## Abstract

**Background:**

Malaria is ranked as the major public health problem in Cameroon, representing 50% of illness in less than five year old children, 40-45% of medical consultation and 40% of the annual home income spent on health. The Cameroon Oil Transportation Company (COTCO) that exploits the Chad-Cameroon pipeline in Cameroon territory, initiated in 2010, a public private partnership project to control malaria along the pipeline corridor. A research component was included in the project so as to guide and evaluate the control measures applied in this pipeline corridor. This study presents the baseline socio-anthropological data as well as the knowledge, attitudes and practices of the local population concerning malaria, its transmission, management and prevention.

**Methods:**

A descriptive cross-sectional survey was undertaken in four sentinel sites (one site per ecological zone) along the Chad-Cameroon pipeline corridor. Three structured questionnaires were used for the survey. Two of them were addressed to the heads of households (one for census and the other to collect information concerning the characteristics of houses and living conditions in households as well as their knowledge, attitudes and practices concerning malaria). The last questionnaire was used to collect information on malaria management and prevention. It was addressed to women who had delivered a living child within the past three years. Interviewers were recruited from each village and trained for two consecutive days on how to fill the different questionnaires. All data were analysed at 5% significant level using Epi-Info, SPSS and Cs PRO 4.0 STATA. Values of p ≤ 0.05 were considered statistically significant.

**Results:**

Interviews were conducted in 2597 households (Bipindi 399, Bélabo 835, in Meidougou 820 and Dompta 543). Whatever the study site, 50% of the heads of household were workers of the agro-pastoral sector. Most of the heads of household were men (average 77.4% for men and 22.6% for females). The walls of households were mostly made-up of earth blocks and access to media was low. There were significant differences between mean ages and educational level of the heads of household. Significant differences were also observed between the characteristics of houses and the sites located in the southern regions (Bipindi and Bélabo) and those located in the northern regions (Meidougou and Dompta). The later household heads were younger and less educated than those in the other regions.

In most of the study sites, paracetamol was cited as the first intention drug for malaria treatment, followed by chloroquine, a banned drug. More than half of the households studied had a correct knowledge of malaria and its mode of transmission: 120/155 (77.1%) in Bipindi, 244/323 (74.5%) in Bélabo, 171/235 (72.8%) in Meidougou and 118/218 (54.1%) in Dompta. Fever and headache were the malaria signs/symptoms most often cited by the households. An important percentage of pregnant women did not take any malaria prophylaxis during their last pregnancy (up to 43.4% in Bélabo).

**Conclusion:**

In all the study sites, there were conditions that indicated the all year round transmission of malaria (characteristics of houses and limited access to media making sensitization campaigns difficult). In general, most households had a good knowledge of malaria and its mode of transmission. However, malaria treatment drugs were most often inappropriate. In this study, recommendations were made in order to guide the implementation of control measures.

## Background

Despite decades of control efforts, malaria remains a major cause of morbidity and mortality in tropical and sub-tropical regions of the world [1]. There are an estimated 300–500 million clinical diseases reported yearly with 90% of them from sub-Saharan Africa, which has an estimated 1–3 million deaths annually [1]. Malaria ranks first as the public health problem in Cameroon, representing 50% of illness in less than five years of age. It also accounts for 40-45% of medical consultations and 40% of annual home income spent on health [2]. The burden of malaria perpetuates poverty and reduces chances of Cameroon government to attain the millennium goals. The Cameroon National Malaria Control Programme of the Ministry of Health is based on the fight against the vector by the use of impregnated mosquito nets, the appropriate management of malaria cases using artemisinin based combination therapy (ACTs) and communication for changing of behaviour. Earlier studies conducted in 2006 showed that only 13% of children below five years were sleeping under impregnated bed nets and that 37% of pregnant women took correct intermittent preventive treatment (IPT) during their last pregnancy. In the same study, it was also observed that 26.2% of simple malaria cases were properly managed using artemisinin based combination therapies [3]. The Cameroon Oil Transportation Company (COTCO) that exploits the Chad-Cameroon pipeline along the Cameroon territory, Initiated in 2010, a public private partnership project to control malaria along the pipeline corridor. In order to attain the objective of this study, some partners were involved in the project. These were the Ministry of Health/National Malaria Control Programme beneficiary of the project, Cameroon Oil Transportation Company (COTCO) provider of logistics (transportation, lodging, etc.), “Exxon Mobil” for financial support, the University of Yaoundé 1 and the University of Bordeaux 2 responsible for the operational research component, “SANOFI AVENTIS” drug manufacturing company provider of anti-malarial drugs for case management, “Service Medical International” (SMI) provider of malaria kit for community use, and the “Association Camerounaise pour le Marketing Social” (ACMS) responsible for the implementation of control measures (impregnated mosquito nets and malaria kits distribution and training of community health workers (CHW). A research component was included in the Chad-Cameroon pipeline project in order to guide and evaluate the control measures applied along the pipeline corridor. This consisted of epidemiological, entomological and socio-anthropological components. This paper presents the baseline socio-anthropological data as well as the knowledge, attitudes and practices of the local population studied concerning malaria, its transmission, management and prevention.

### General objective

To identify socio-anthropologic factors that affects the persistence of malaria and determines the knowledge, attitudes and practices of the population, concerning malaria and its transmission.

### Specific objectives

–To identify human and environmental factors that favour malaria endemicity,

–To evaluate knowledge, attitudes and practices of heads of households as concerns malaria,

–To identify mother’s behaviour regarding the management of febrile episodes in infants/children,

–To formulate recommendations that will be used as guidelines in the implementation of control measures.

## Methods

### Study design

This was a cross-sectional, prospective and analytical study.

### Study sites

**Figure 1:**
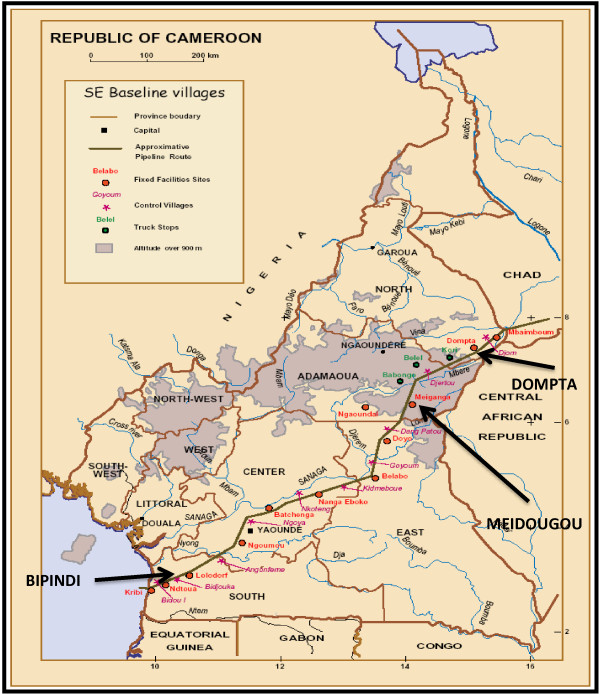
**Study sites along the Chad-Cameroon pipeline (Source: COTCO archives).** LEGEND. Study sites.

The Chad-Cameroon pipeline crosses 234 villages from Ebomé (Atlantic Ocean) to Mbaï-Mboum (Chad-Cameroon border) and covers a distance of 890 kilometers going through a variety of physical environments (tropical evergreen forest, forest-savannah, and savannah). These physical environments have been divided into four ecological zones in this study. Four sentinel sites were chosen according to ecological zone (Figure 1).

#### Study site N°1

Bipindi health area has a population estimated at 2,570 inhabitants. Hunting, fishing and farming are the main activities of this area. It is located in the South Region of Cameroon near the western coast of Africa in the Atlantic littoral evergreen forest (10°28′E, 3°07′N). Three villages (Bipindi rural, Bidjouka and Bikalla) were selected in this study site. Bipindi health area is in the humid forest ecological zone with a mono-modal rainfall climate. The annual rainfall ranges from 2,000 to 10,000 mm, being very high where Cameroon’s volcanic massif comes close to the coast. In general, the highest rainfall occurs between the months of July and September ranging from 400 to 500 mm and declines from about 400 mm, in the wet season, to about 100 mm.

An annual average temperature of 23-25°C has been recorded.

#### Study site N°2

Bélabo health area is found in the East Region which is in the humid dense forest ecological zone of Cameroon (13°18′E, 4°56′N). It is a town lying on the Yaoundé-Ngaoundéré railway line. Bélabo health area is covered by many rivers including Sesse, Lom, Pangar, Doh yon and the Sanaga The population is estimated at 4,330 inhabitants. Hunting, fishing and farming are the main activities of this area. Six villages (Ndoumba Kanga, Bélabo, Essandjané, Ebaka I, Yébi and Biombé) were selected in this study site. The climate is equatorial with a bimodal rainfall. It has two dry seasons (July to August and mid-November to mid-March) coupled with two rainy seasons (August to mid-November and mid-March to June). An annual average temperature of 30°C has been recorded.

#### Sentinel site N°3

Meidougou health area is located in the Adamawa Region of Cameroon (14°13′E, 6°25′N) and classified in the high guinea savannah ecological zone. This zone is dominated by trees and bush savanna found in the highland plateau of Adamawa Region.

The population is estimated at 3,190 inhabitants. They are merchants, farmers and manage cattle ranches. The climate is tropical which is characterized by two seasons. Annual rainfall varies between 1700 and 3000 mm. Three villages (Dankali, Meidougou and Bounou) were selected in this study site.

#### Sentinel site N°4

Dompta health area, found in the North Region of Cameroon, is situated 100 kms away from Touboro, headquarter of the health district (15°09′E, 71°18′N). The population is estimated at 1,000 inhabitants. They are farmers and manage cattle ranches. It is in the sudana-sahelian ecological zone which is made up of the Mandara Mountains, the far north plains and the Benue valley. The annual rainfall ranges from 800 to 900 mm, from the month July to October while the remaining eight months are dry. There is a dense hydrographic network made up of dry river beds (locally called Mayos) and permanent rivers. This zone is at the risk of desertification. Three villages (Mboko, Bougoui and Bemboyo) were selected in this study site.

### Study duration

Data were collected during a week in each of the four study sites. This was from the month of September 2010 for Meidougou and Dompta sites, December 2010 for Bipindi site and March 2011 for Bélabo site.

### Study population

This was made-up of heads of household or their representatives and women who had given birth to a child during the past three years.

#### Inclusion criteria

The inhabitants included in the study were those who had been residents in the village concerned since at least six months and those who had given their informed consent to participate into the study.

#### Exclusion criteria

The inhabitants excluded in this study were those with uncompleted questionnaires and those who had withdrawn their informed consents.

### Sampling method

In each village of the study sites, households were randomly chosen to participate in the interviews. This was initiated from chief of village’s house and then moved from east to west, as well as, from the north to south in the following manner. For a population ≤ 1000 inhabitants, one out of two households were included while for a population ranging from 1,001 to 2,000 inhabitants, one out of three households were included. For a population of more than 2,000 inhabitants, one out of four households was selected to participate in this study.

### Procedures

Before each field collection of data, the interview schedule was sent to the different village chiefs and community health workers concerned. This was to permit them to start sensitizing the population before the arrival of interviewers. On arrival in each village, the administrative and traditional rulers were met in order to explain the aim and purpose of the visit and to obtain their different authorizations.

#### Filling of questionnaires

The interviewers, holders of at least a First School Leaving Certificate (Primary education) were recruited from each village with the help of community health workers. In addition, the interviewers were expected to speak and write the French language as well as speak the local language. The selected interviewers were trained for two consecutive days on how to fill the questionnaires and on good behaviour during interviews. Each interviewer received a code and was asked to write it on all completed questionnaires and on the main door of each corresponding household where he/she had carried out the interview. This was to prevent duplication by another interviewer. Three types of questionnaires were used in this study:

#### Questionnaire N°1

This was addressed to heads of households and served the purpose of a census, (list of people living in the household, their names, ages, sex, educational level and their relationship with the head of the household.

#### Questionnaire N°2

This questionnaire was also addressed to heads of households. It was used to collect information on the living conditions in households, their knowledge, attitudes and practices about malaria.

#### Questionnaire N°3

The last questionnaire was used to collect information on malaria management and prevention. It was addressed to women who had delivered a living child within the past three years.

### Ethical considerations

An ethical clearance was obtained from the Cameroon National Ethical Committee (Authorization N° 040/CNE/SE/2010 of 18/12/2010). A letter of information was given to each selected household to read. The interviewer read the letter of information in the French language or translated it in to the local language. Households willing to participate in the study signed on a consent form. For those unable to write their finger print on the consent form served the purpose. However, households were free to withdraw from the study at any moment without any explanation. All information obtained from the households was treated as confidential.

### Data analysis

Field data were checked for validity and entered into the computer using Microsoft Excel 2007 and Access software. Data were analyzed at 5% significant level using Epi-Info, SPSS Statistical packages, and Cs PRO 4.0 STATA. Values of p ≤ 0.05 were considered statistically significant.

## Results and discussion

### Results

#### Socio-demographic characteristics

Data obtained from the study showed that interviews were conducted in 2,597 households while the census indicated 14,216 inhabitants. Bipindi study site had 399 households with 2565 inhabitants, Bélabo study site had 835 households with 4,429 inhabitants, Meidougou study site had 820 households with 4,027 inhabitants while Dompta study site had 543 households with 3,195 inhabitants.

a.) Mean age of heads of households

The mean ages (Table 1) were higher in the study sites located in southern region (Bipindi 52 ± 16.5 yrs. and Bélabo 41 ± 18.9 yrs.) than those in the northern regions (in Meidougou 24 ± 2.75 yrs. and in Dompta 34 ± 13.4 yrs.).

b.) Sex distribution of household heads

In most of the study sites, the majority of heads of households were men (52.2% in Bipindi, 84.4% in Bélabo, 74% in Meidougou and 89.7% in Dompta). The results obtained showed that men (77.3%) were more represented in the households than women (22.7%).

c.) Distribution of ethnic groups per study site

In Bipindi, the two main ethnic groups were the Ngoumba (60%) and the Bassa (26%) while in Bélabo, the Bobilis ethnic group (88.4%) was the most represented. In the two northern study sites, the ethnic groups were the same. However, their distribution varied from one study site to the other. In Meidougou, 57.2% heads of households belonged to the Baya tribe whereas in Dompta, the Mboum tribe was dominant (42.2% of heads households belonged to this tribe).

d.) Matrimonial status

In all the study sites, the majority of heads of households were married (Bipindi 48%, Bélabo 48.9%, Meidougou 65.7% and Dompta 77.2%) with an average of 59.6% (p = 0.000). The percentage of married heads of households increased from Bipindi study site in the south region to Dompta study site in the north region. On the contrary, the percentage of free union of couples decreased from Bipindi to Dompta (Bipindi, 26.9%, Bélabo 16.7%, Meidougou 2.4% and Dompta 0.8%).

e.) Religion

It was observed that Christianity was predominant in three study sites (Bipindi 98.2%, Bélabo 90.2%, and Dompta 64.3%). On the contrary, Islam was predominant in Meidougou (49.5%).

f.) Educational level

In this study, the percentage of heads of households (Table 2) who had no formal education increased from the study sites located in the humid forest ecological zone (Bipindi 13.3% and Bélabo 20.6%) to those found in the savannah ecological zone (Meidougou 51.1%) and to the study site located in the Sahel region (Dompta 54.2%). On the other hand, the percentage of heads of households who had attended secondary school decreased from the southern (Bipindi, 40.7% and Bélabo 22.4%) to the northern regions (Meidougou 14.7% and Dompta 18.9%).

g.) Profession of heads of households

In most of the study sites, at least 50% of heads of households were workers of the agro-pastoral sector (Bipindi 53.6%, Bélabo 60.1%, Meidougou 70.5% and Dompta 65.8%).

**Table 1:** Distribution of household heads according to their ages

**Age (years)**	**Sites**				**TOTAL**
	**Bipindi N (%)**	**Bélabo N (%)**	**Meidougou N (%)**	**Dompta N (%)**	**N (%)**
<20	1 (0.2)	46 (5.5)	59 (8)	20 (4)	126 (5.1)
[20-30[	24 (6.3)	187 (22.4)	197 (26.5)	110 (22.1)	518 (21.1)
[30-40[	63 (16.6)	217 (26)	146 (19.5)	131 (26.3)	557 (22.6)
[40-50[	88 (23.2)	137 (16.4)	119 (16)	75 (15.1)	419 (17)
[50 & above [	203 (53.5)	247 (29.6)	171 (23)	71 (14.3)	692 (28.1)
Do not know	(0)	1 (0.1)	53 (7)	91 (18.3)	145(5.9)
Mean age	52 ± 16.5	41 ± 18.9	24 ± 2.75	34 ± 13.4	37.74 ± 12.88
Total	379 (100)	835 (100)	745 (100)	498 (100)	2457 (100)

**Table 2:** Educational level of households heads

**Educational level**	**Bipindi**	**Bélabo**	**Meidougou**	**Dompta**	**TOTAL**
	**N (%)**	**N (%)**	**N (%)**	**N (%)**	**N (%)**
No level	51 (13.3%)	172 (20.6%)	380 (51.1%)	270 (54.2%)	873 (35.6%)
Primary	174 (46%)	470 (56.3%)	257 (34.5%)	134 (26.9%)	1035 (42.2%)
Secondary and above	154 (40.7%)	187 (22.4%)	108 (14.7%)	94 (18.9%)	543 (22.1%)
TOTAL	379 (100%)	829 (100%)	745 (100%)	498 (100%)	2451 (100%)

### Characteristics of the habitats and living conditions in the households

a.) Information concerning the habitats

Four criteria were considered in their evaluations which are the roof, the ceiling, the walls and the presence of mosquito nets on windows. Study sites located in the southern regions had the majority of their houses roofed with corrugated sheets (Bipindi 95.5% and Bélabo 76.1%) (Table 3). In the Savannah and Sahel regions (Meidougou 58% and Dompta 90%) the roofs were made up of straw. The presence of ceilings in the houses was rare in Bipindi (13.6%) and Bélabo (10.7%). On the contrary, ceilings were present in Meidougou (66%) and Dompta (84%) study sites. As regards the walls, they were mostly made-up of earth in whatever the study site. The presence of nets on the windows was rare in the houses of study sites located in the forest ecological zones (Bipindi 2.2% and Bélabo 11%). In the northern study sites, more than 30% of the houses had mosquito nets on their windows.

**Table 3:** Characteristics of houses per site

**Sites**		**Bipindi**	**Bélabo**	**Meidougou**	**Dompta**	**TOTAL**
		**N = 155**	**N = 319**	**N = 275**	**N = 150**	**N = 899**
Roofs	- Zinc (metal sheets)	148 (95.48%)	248 (76.1%)	77 (28%)	6 (4%)	479 (53.28 %)
	- Straw	4 (2.58%)	22 (6.7%)	160 (58%)	136 (90%)	322 (35.81 %)
	- Raffia	3 (1.94%)	49 (15%)	38 (14%)	9 (6%)	99 (11.01 %)
Ceiling present		21 (13.55 %)	35 (10.70%)	180 (66%)	131 (84%)	367 (40.82%)
Walls	- Mud	99 (63.87%)	227 (69.60%)	275 (100%)	150 (100%)	751 (83.53 %)
	- Semi cement	33 (21.29%)	35 (10.7%)	0	0	68 (7.56 %)
Windows with nets (%)		3 (2.21 %)	36 (11%)	101 (35.8%)	56 (36.1%)	196 (21.80%)

b.) Presence of electricity and exposure to media

In all the study sites, the presence of electricity was rare. The highest percentage of households using electricity was observed in Meidougou (39%). In general, more than 40% of households had a radio in all the study sites except in the Bélabo study site where only 28.3% had this communication tool. During this study, television sets were found in of homes in Bipindi (18.06%), in Bélabo (23.30%), Meidougou (6.50%) and less in Dompta (0.6%) households. Except in Bélabo where 51.2% households had a mobile phone, this communication tool was used by less than 50% of households in all the other study sites. However, the signal was most often weak.

### Knowledge, attitudes and practices concerning malaria

a.) General knowledge of malaria

In all the study sites, more than half of the households had a correct knowledge of malaria and its mode of transmission [Bipindi 120/155 (77.08%), Bélabo 244/323 (74.5%), Meidougou 171/235 (72.8%) and Dompta 118/218 (54.1%)]. Fever and headache were the signs/symptoms most often cited by the households whatever the study site and convulsions and diarrhea were the less cited (Table 4).

b.) Management of fever and malaria

**Figure 2:**
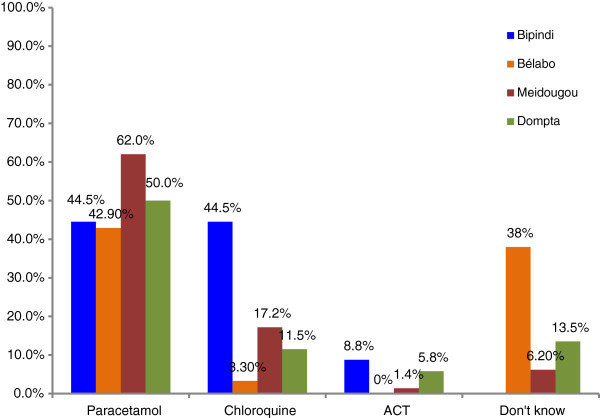
Drugs used for malaria treatment.

Mothers were asked if one of their children had fever/malaria during the last two weeks before the interview. More than 1/3 of the mothers answered positive while a majority sort for appropriate care within 48 hrs from the onset of fever. In most of the study sites, paracetamol was cited as the first intention drug for malaria treatment followed by chloroquine which is a banned drug in Cameroon. Only a few mothers from Bipindi (8.8%), Dompta (5.8%) and Meidougou (1.4%) knew about artemisinin based combination therapies (ACTs), the drug combinations recommended by the Ministry of Health (Figure 2). The places from where malaria drugs were purchased varied from one study site to another. Except for Dompta where 65% of households purchased drugs from inappropriate places (mostly from shops), in all the three other study sites, drugs were purchased from private pharmacies and health institutions. Traditional healers and community health workers were rarely cited as drug providers.

**Table 4:** Knowledge of signs and symptoms of malaria

**Sites**					
**Nb (%)**					
**Signs/symptoms**	**Bipindi**	**Bélabo**	**Meidougou**	**Dompta**	**TOTAL**
N	155	323	235	218	931
Fever	43 (27.7%)	263 (80.7%)	48 (20.3%)	53 (24.1%)	407 (43.7%)
Headache	41 (26.4%)	235 (72.1%)	52 (21.9%)	48 (22%)	376 (40.3%)
Anorexia	17 (11.1%)	237 (72.7%)	13 (5.4%)	13 (6%)	280 (30%)
Vomiting	16 (10.1%)	205 (62.9%)	36 (15.5%)	21 (9.5%)	278 (29.8%)
Convulsions	1 (0.7%)	73 (22.4%)	4 (1.5%)	5 (2%)	83 (8.9%)
Diarrhea	2 (1.5%)	73 (22.4%)	6 (2.5%)	5 (2.5%)	85 (9.1%)
Joint pains	16 (10.3%)	113 (34.7%)	24 (10.2%)	32 (14.7%)	185 (19.8%)
Sweating/Shivering	-	137 (42.41%)	35 (15%)	29 (13.3%)	201 (21.5%)

c.) Malaria prevention

This study showed that knowledge on the use of impregnated mosquito nets was very high in all the study sites (Bélabo 75.5%, Bipindi 90.3%, Meidougou 90.4% and Dompta 97.4%). As one goes from the southern study sites to the northern ones, the percentage of households owning at least an impregnated mosquito bed net increases (from Bipindi 37.9% to Dompta 75.2%). In addition, the households studied (Bipindi 62.7%, Bélabo 80.9%, Meidougou 55.7% and Dompta 72.4%) reported that the main advantage of using an impregnated mosquito bed net was protection against mosquito bites. Hence, the protection against malaria and other diseases was reported by households (Bélabo, 65.1%, Meidougou 42.6%, Bipindi 37.2% and Dompta 27.1%). The three disadvantages of impregnated mosquito bed nets frequently reported by the households were heat, feeling of being suffocated and high cost. Whatever the study site, an important percentage of pregnant women did not take any prevention against malaria during their last pregnancy (Bélabo 43.4%). However, it was observed that some of women continue to take chloroquine (Bipindi 50%). The percentage of women who had taken, at least, one dose of recommended Sulfadoxin-pyrimethamin Oral (*give in full*) is low, ranging from 19.5 to 50%.

### Discussion

#### Limitations of the study

During this study, due to transportation problems during the rainy season and the nature of the roads at this period of the year, certain field trips had to be rescheduled. In addition, the questionnaires did not contain a section to determine the correctness of the local language translation given by the interviewers. This shortcoming could have introduced a bias in the data collected.

#### Socio-demographic characteristics

In this study, 2597 heads of households were interviewed in the four selected study sites. Earlier studies used sample sizes that varied from one study to the other [4-8]. The mean ages of the households varied from 24 years in Dompta to 52 years in Bipindi. In the study sites located in the northern region of Cameroon, the heads of households were younger than in the sites located in the southern region. This variability in the mean ages could be due to differences in the local culture of the heads of households [9]. It was observed that most of the household heads were men. This observation corroborates the results obtained from the National Demographic and Health Survey which indicated that 76% of heads of Cameroonian households were men [9,10]. Similar results have also been reported in previous studies [4,8]. In Bipindi and Bélabo study sites, less than 50% of the heads of households were married while in the northern regions (Meidougou and Dompta study sites) more than 60% of the heads of households were married. The discrepancy between the southern and northern regions could be explained by the fact that generally in the south, the Bantu and semi-Bantu cultures are predominant while in the northern regions it is the Sudanese and the Hamite culture [11]. In previous studies, the proportion of heads of households who were married varied from one study site to another with respect to the local culture [4,5,12]. In all the survey sites, most of the heads of households carried out an agro-pastoral activity. This may be due to the fact that the study was conducted in rural zones and the households were sedentary. Other studies carried out in rural zones have also showed that agro-pastoral activities were dominant in this type of setting [4,5,13]. In the Bipindi study site, Ngoumba and Bassa were the majority ethnic groups while in the Bélabo study site; the Bobilis were the dominant tribe. However, in Meiganga and Dompta study sites, it was the Baya, the Fulbé and the Mboum ethnic groups. These observations correspond to the distribution of ethnic groups in Cameroon [10]. These local languages could be used in planning sensitization messages. The level of educational is an important factor which contributes to the improvement of living conditions and it affects procreative behaviour, health and hygienic habits of a population [14]. It was observed that the percentage of illiterate heads of households increases from the south to the north region. The North Regions had the highest level of illiteracy (Meidougou 51.0% and Dompta 54.2%). This result corroborates those reported in the National Demographic and Health Survey [9] and indicates that the Northern Region of Cameroon has the least level of education despite the government efforts.

#### Characteristics of the habitats and living conditions in the households

Most of the roofs of households in Bipindi and Bélabo study sites were made up of corrugated sheets while those of Meidougou and Dompta study sites were of straw. Ceilings were most often absent in Bipindi and Bélabo study site houses while it was present in Meidougou (66%) and Dompta (84%) houses. The majority of walls were of earth blocks which do not permit the retention of insecticides usually used [15,16]. In all the study sites, it was observed that the characteristics of the habitats favored human-mosquito contact. The highest percentage of electricity supply was noted in Meidougou (39%) and Bipindi (29%) households. Except for Dompta, where only 15% of households had electricity, in the other study sites, the percentage of households (16%) that had electricity was higher than those reported for rural zones in Cameroon [9]. Data concerning exposure to the media are important for the broadcasting of information and educative programs. The presence of a TV set in households varied from 0.6% in Dompta to 23.3% in Bélabo. However, 28 to 56% of households had radios though the signals were weak. Mobile phones were also present in 29 to 51% of the households. In order to reach the maximum of people in the community, sensitization programs on the fight against malaria or any other disease could combine many communication tools such as radio spots, mobile phone messages, focus group discussion using French and local languages.

#### Knowledge, attitudes and practices concerning malaria

a.) General knowledge of malaria

In this study, most of the households had a good knowledge of malaria and its transmission and they (653/931; 70.1%) correctly associated malaria to mosquito bites. These results corroborate those reported for a rural zone in Swaziland [4]. However, they are low when compared to those reported (99 and 75.9%) for Douala, the biggest city in Cameroon [17,18]. In Yaoundé, the capital city of Cameroon, the percentage was also higher (88.8%) than those found in the present study [18]. These differences are probably due to the fact that Douala and Yaoundé are big cities whereas this study was carried out in rural areas. In an urban community of south western Nigeria, 93.2% of households recognized mosquito bites as the cause of malaria [19]. In the present study, knowledge of malaria was independent of the level of education. This observation differs from that obtained in Ndu community of the North West region of Cameroon, where it was reported that the level of education was a significant indicator of malaria knowledge [20]. The most cited signs and symptoms of malaria were fever and headache regardless of the study site. This is consistent with those of a study conducted in Swaziland [4].

b.) Malaria management and prevention

It was observed that the time lapse between the onset of fever in children and that to seek care by mothers was 48 hours. However, this is an acceptable time lapse though the ideal should be 12–24 hours [21]. In all the study sites, the therapeutic means used to treat malaria were not always appropriate. Paracetamol was the first intention drug followed by chloroquine. Though the later was banned in the Cameroonian market, since 2002, many households declared that they still used it both for malaria treatment and prevention. However, artesunate-amodiaquine, the ACT recommended by the MoH, is used by very few people. It was also observed that the behavior of in households depended on the closeness of their homes to health facilities. Households living near a health facility had the tendency to buy antimalarial drugs from this structure whereas those living far away had the tendency to go to the nearest shops and street vendors. The percentage of households that own at least one impregnated mosquito net increased gradually from the south to the north. In Meidougou study site, due to the presence of many refugee camps, several NGOs have included in their activities the distribution of impregnated mosquito net which is often also distributed to the local population. In Dompta, one of Cameroon Oil Transportation Company’s that has pumping stations, impregnated mosquito nets are distributed to workers and to the entire community. However, there exist some myths about impregnated mosquito nets that limit its use (heat under the nets, feeling of being suffocated, etc.). These ideas should be removed from sensitization campaigns. Since 2004, the Cameroon government has adopted sulfadoxin-pyrimethamin (SP) for the intermittent preventive treatment of malaria in pregnant women. Chloroquine, a banned drug, was used by almost 50% of pregnant women for malaria prevention. In addition, 30 to 60% of women did not receive any malaria prevention. It was reported that 47% of pregnant women received drugs for malaria prevention during their last pregnancy, but the distribution rates varied from one zone to another [9]. In the Center and South regions, up to 70% of pregnant women took malaria prevention against 32% in the Adamawa and North regions. In the present study, the same pattern of distribution of women who received malaria prevention during their last pregnancy was observed.

## Conclusions

In this study, it was observed that there is a discrepancy in almost all the indicators (ethnic groups distribution, ages and the level of education of heads of households, characteristics of the households, etc.), between the study sites located in the southern region (Bipindi and Bélabo) and those located in the northern regions (Meidougou and Dompta).

From the results obtained, the following recommendations were made in order to serve as a guide in the control of malaria:

–that sensitization programs on the fight against malaria or any other disease could combine many communication tools such as radio spots, mobile phone messages, focus group discussions using French, the official language in the study sites and local languages of the major ethnic groups,

–that sensitization messages should insist on the drugs recommended by the MoH for malaria treatment and prevention,

–that along the pipeline corridor, indoor spraying should not be used as an anti-vectorial control method,

–that during sensitization campaigns, the myth about impregnated mosquito nets that limit its use (heat under the nets, feeling of being suffocated, etc.). These ideas should be rolled out

## Competing interests

The authors declare that they have no competing interests.

## Authors’ contributions

RMS, PE, PM and ES planned the study design. RMS, ES, JPK, NNT, HNH and AN performed field activities. ES and RMS drafted the manuscript. All authors read and approved the final manuscript.

## Pre-publication history

The pre-publication history for this paper can be accessed here:

http://www.biomedcentral.com/1471-2458/13/1023/prepub
